# Personality Factors' Impact on the Structural Integrity of Mentalizing Network in Old Age: A Combined PET-MRI Study

**DOI:** 10.3389/fpsyt.2020.552037

**Published:** 2020-11-17

**Authors:** Panteleimon Giannakopoulos, Cristelle Rodriguez, Marie-Louise Montandon, Valentina Garibotto, Sven Haller, François R. Herrmann

**Affiliations:** ^1^Department of Psychiatry, University of Geneva, Geneva, Switzerland; ^2^Medical Direction, Geneva University Hospitals, Geneva, Switzerland; ^3^Department of Readaptation and Geriatrics, Geneva University Hospitals and University of Geneva, Geneva, Switzerland; ^4^Division of Nuclear Medicine and Molecular Imaging, Diagnostic Department, Geneva University Hospitals, Geneva, Switzerland; ^5^Faculty of Medicine of the University of Geneva, Geneva, Switzerland; ^6^CIRD - Centre d'Imagerie Rive Droite, Geneva, Switzerland; ^7^Department of Surgical Sciences, Radiology, Uppsala University, Uppsala, Sweden

**Keywords:** amyloid load, cohort studies, mentalizing, personality, structural MRI

## Abstract

The mentalizing network (MN) treats social interactions based on our understanding of other people's intentions and includes the medial prefrontal cortex (mPFC), temporoparietal junction (TPJ), posterior cingulate cortex (PCC), precuneus (PC), and amygdala. Not all elders are equally affected by the aging-related decrease of mentalizing abilities. Personality has recently emerged as a strong determinant of functional connectivity in MN areas. However, its impact on volumetric changes across the MN in brain aging is still unknown. To address this issue, we explored the determinants of volume decrease in MN components including amyloid burden, personality, and APOE genotyping in a previously established cohort of 130 healthy elders with a mean follow-up of 54 months. Personality was assessed with the Neuroticism Extraversion Openness Personality Inventory-Revised. Regression models corrected for multiple comparisons were used to identify predictors of volume loss including time, age, sex, personality, amyloid load, presence of APOE epsilon 4 allele, and cognitive evolution. In cases with higher Agreeableness scores, there were lower volume losses in PCC, PC, and amygdala bilaterally. This was also the case for the right mPFC in elders displaying lower Agreeableness and Conscientiousness. In multiple regression models, the effect of Agreeableness was still observed in left PC and right amygdala and that of Conscientiousness was still observed in right mPFC volume loss (26.3% of variability, significant age and sex). Several Agreeableness (Modesty) and Conscientiousness (order, dutifulness, achievement striving, and self-discipline) facets were positively related to increased volume loss in cortical components of the MN. In conclusion, these data challenge the beneficial role of higher levels of Agreeableness and Conscientiousness in old age, showing that they are associated with an increased rate of volume loss within the MN.

## Introduction

Mentalizing is the term used to qualify brain ability to treat social interactions that rely on our understanding of other people's intentions, beliefs, traits, and other high-level characteristics [for a review, see (1)]. Early behavioral research showed that such social inferences are mostly made implicitly without any cognitive control (2). Later event-related potential and functional magnetic resonance imaging (MRI) contributions have started to uncover the key brain regions supporting our ability to “think about other's mental states,” giving rise to what has become known as the mentalizing network. The main components of this network are the medial prefrontal cortex (mPFC), temporoparietal junction (TPJ), posterior cingulate cortex (PCC), and precuneus (PC) and less commonly the amygdala [for reviews, see (3–7)]. Although the exact role of its area in the construction of social cognition is not yet elucidated, the most widely accepted hypothesis is that social inferences are hierarchically arranged with amygdala providing valenced information, TPJ interpretation of behaviors, PCC and PC imagery and imagination processes needing to infer the metal states of another, and mPFC final interpretations in terms of intentions and traits (1, 5, 8).

Personality patterns affect activation of the mentalizing network. For instance, avoidant traits were associated with higher task-related activation of mPFC, amygdala, and cingulate cortex, whereas the inverse was true in persons with high levels of Neuroticism (9). In the same line, extraversion was negatively associated with PC functional connectivity (10). In addition, personality has recently emerged as a strong determinant of functional connectivity in DMN that includes most of the key areas of the mentalizing network. In particular, high level of openness to experience but also mind wandering were positively related to DMN functional connectivity (11–14). In contrast, both positive and negative associations were reported between this variable and Agreeableness facets. In particular, the connectivity between PCC and PC decreased in cases with high honesty facet (15).

Old age is known to affect theory of mind performances related to both mentalizing during person perception and in virtual settings in the absence of neurodegenerative disorders (4, 16, 17). Lower activity in mPFC and PC has been reported across a variety of social cognitive tasks in healthy elders (18, 19). Importantly, the mentalizing network key nodes are parts of the default mode network (DMN) that displays well-known age-related disturbances of its structural and functional connectivity [for a review, see (20)]. A recent study postulated that AD risk was associated with DMN gray matter volume loss in elderly controls over 60 years of age (21). Not only functional but also volumetric changes in the mentalizing network may affect its performances in old age. Surprisingly, in contrast to functional MRI observations, the aging-related volumetric changes across the mentalizing network and their determinants have been rarely investigated in longitudinal settings. Only two cross-sectional contributions reported aging-related volume loss in mPFC, PC, and PCC and pointed to an increased rate of 1-year atrophy that partly matched the frontotemporal pattern of changes in healthy aging (22, 23).

Whether or not personality factors accelerate or prevent age-related changes in mentalizing network is still unknown. We report here the data from a longitudinal analysis exploring the determinants of volume decrease in mentalizing network components including amyloid burden, personality, and APOE genotyping in a previously established cohort of 130 healthy elders with a mean follow-up of 54 months. Based on the previously cited observations on the relationship between personality factors and mentalizing network connectivity, we hypothesized that Openness to experience and Agreeableness, two of the five personality factors identified by the Big-Five/Five-Factor model (24), may decrease the aging-related volume loss in mentalizing network.

## Methods

### Population

The selection of cases among participants of a still ongoing cohort study was described in detail in our recent contribution focusing on the effect of personality in memory-related areas (22). Briefly, all of the cases were recruited via advertisements in local newspapers and media. Exclusion criteria included psychiatric or neurologic disorders; sustained head injury; history of major medical disorders (neoplasm or cardiac illness); alcohol or drug abuse; regular use of neuroleptics, antidepressants; or psychostimulants; and contraindications to PET or MRI scans. To control for the confounding effect of vascular pathology on MRI findings, individuals with subtle cardiovascular symptoms, hypertension (non-treated), and a history of stroke or transient ischemic episodes were also excluded from the present study. The initial cohort included 526 elderly white non-Latinos of mixed European descent individuals living in Geneva and Lausanne catchment area. Due to the need for an excellent French knowledge (in order to participate in detailed neuropsychological testing), the vast majority of the participants were Swiss (or born in French-speaking European countries, 92%). Cases with three neurocognitive assessments at baseline, 18 months, and 54 months; structural brain MRI at baseline and 54 months post-inclusion; brain amyloid PET at follow-up; and APOE status were considered. The sample 54 months post-inclusion included 397 cases (25–28). As a sub-project of this cohort study, the NEO-PI-R assessment was administrated randomly at inclusion in 130 elderly controls (Table 1).

**Table 1:** Clinical, demographic, and PET data according to the amyloid status in the present series.

	**PET amyloid**			***P*-values**
	**Negative**	**Positive**	**Total**	
*N*	84	46	130	
Female	60 (71.4%)	22 (47.8%)	82 (63.1%)	0.013
Age at Amy PET	79.5 ± 4.4	78.8 ± 3.6	79.3 ± 4.1	0.336
Education (year)				0.065
<9	16 (19.0%)	2 (4.3%)	18 (13.8%)	
9–12	40 (47.6%)	24 (52.2%)	64 (49.2%)	
>12	28 (33.3%)	20 (43.5%)	48 (36.9%)	
APOE4 positive	8 (9.5%)	16 (34.8%)	24 (18.5%)	0.001
MMSE at baseline	28.4 ± 1.3	28.8 ± 1.0	28.6 ± 1.2	0.089
Change in cognition	−0.2 ± 3.5	−2.3 ± 4.0	−0.9 ± 3.8	0.004
Neuroticism (N)	79.9 ± 17.5	75.6 ± 20.7	78.4 ± 18.7	0.234
Extraversion €	99.4 ± 15.0	100.7 ± 15.6	99.8 ± 15.2	0.647
Openness (O)	113.5 ± 16.6	109.3 ± 17.2	112.0 ± 16.9	0.181
Agreeableness (A)	132.8 ± 16.8	125.3 ± 18.5	130.2 ± 17.7	0.025
Conscientiousness ©	115.2 ± 16.3	111.3 ± 16.1	113.8 ± 16.3	0.196
Mean SUVr	0.6 ± 0.1	0.7 ± 0.1	0.6 ± 0.1	<0.001

### Personality Assessment

Personality features and dimensions were assessed at baseline using the French version of the NEO-PI-R (24). Participants completed the 240-item self-report version of the NEO-PI-R questionnaire using a five-point Likert agreement scale. The NEO-PI-R assesses 30 facets, 6 for each of the five personality factors (Neuroticism, Extraversion, Openness, Agreeableness, and Conscientiousness). Neuroticism is the tendency to feel negative emotions including anxiety, hostility, and anger; Extraversion encapsulates the proneness toward positive emotions and feelings such as warmth and enthusiasm; Openness, the personal inclination to experience and the appreciation of new situations and thoughts with a curious, imaginative, and creative attitude, is defined along six facets that cover imagination (or fantasy), sense of aesthetics, emotions, and feelings, but also proactive behaviors and actions to explore and experiment beyond habits and routines, as well as intellectual curiosity, and the disposition to negotiate and discuss social, political, and religious values; Agreeableness is characterized by trustful, cooperative, and altruistic tendencies; and, finally, Consciousness is the predisposition to be reliable, resolute, and well-organized, and unwilling to deviate from rules and moral principles.

### Neuropsychological Assessment

At baseline, all individuals were evaluated with a neuropsychological battery described in detail previously (27, 29–31). All individuals were also evaluated with the Clinical Dementia Rating scale (CDR) (32). According to the criteria of Petersen et al. (33), participants with a CDR of 0.5 but no dementia and a score exceeding 1.5 standard deviations below the age-appropriate mean in any of the cognitive tests were classified as MCI and were excluded. Participants with neither dementia nor MCI were classified as cognitively healthy controls and underwent two additional cognitive assessments after a mean period of 18 and 54 months.

In the absence of consensus, the definition of groups within the normal range on the basis of neuropsychological criteria should avoid to include a priori hypotheses on the cognitive fate of cases with unstable cognitive performances. Among them, some cases progress at the first follow-up and remain stable or even improve their performance at the second follow-up. Others are stable at the first follow-up and progress later on (but may improve or remain stable at later time points). To resolve this difficult question, we calculated the number of tests with improved minus the number of tests with decreased performances resulting in a final continuous cognitive score for each time point. Change in cognition between inclusion and last follow-up was defined as the sum of the continuous cognitive scores at two follow-ups. This new approach makes it possible to avoid a priori hypotheses regarding the longitudinal evolution of cognition in our cases. Cognitive trajectories were defined after summing the number of cognitive tests at follow-up with performances at least 0.5 standard deviation (SD) higher or lower compared with the first evaluation (*Z* scores). Change in cognition between inclusion and last follow-up was defined as the sum of the continuous cognitive scores at two follow-ups as previously described (25, 30).

### Amyloid PET Imaging

One hundred twenty-two 18F-Florbetapir (Amyvid) and 8 18F-Flutemetanol PET (Vizamyl) data were acquired on two PET scans (Siemens BiographTM mCT scanner and GE Healthcare Discovery PET/CT 710 scanner) of varying resolution and following different platform-specific acquisition protocols. The 18F-Florbetapir images were acquired 50–70 min after injection, and the 18F-Flutemetanol PET images were acquired 90 to 120 min after injection. PET images were reconstructed using the parameters recommended by the ADNI protocol aimed at increasing data uniformity across the multicenter acquisitions (22).

Amyloid positivity was visually assessed following standardized procedures approved by the European Medicinal Agency. Moreover, all scans were intensity normalized using the thalamus-pons as target region as described by Lilja et al. (34), and cortical standard uptake value ratios (SUVRs) were then calculated.

### MR Imaging

At baseline, imaging data were acquired on a 3-T MRI scanner (TRIO SIEMENS Medical Systems, Erlangen, Germany). The structural high-resolution T1-weighted anatomical scan was performed with the following fundamental parameters: 256 × 256 matrix, 176 slices, 1 mm isotropic, TR = 2.27 ms. Due to change of MR equipment, follow-up imaging was performed on a 3-T MR750w scanner (GE Healthcare, Milwaukee, Wisconsin) including a high-resolution anatomical 3DT1 sequence (254 × 254 matrix, 178 slices, 1 mm isotropic, TR = 7.24 ms). At both acquisition times, additional sequences (T2w imaging, susceptibility-weighted imaging, and diffusion tensor imaging) were used and analyzed by an experienced neuroradiologist to exclude incidental brain lesions. The average interval between baseline and follow-up imaging was 4.5 ± 0.6 years.

Automatic MR volumetry of both baseline and follow-up MRI was performed with the Combinostics cNeuro software package, using the standard processing parameters as described in the software package (https://www.cneuro.com). Our analysis included both the most frequently cited areas of the mentalizing network (mPFC, TPJ, PC, and PCC) and angular gyrus and amygdala. In order to examine the specificity of our findings, we also analyzed the personality impact on the volume of three control areas (caudate nuclei, fusiform gyrus, and cerebellum). Volume loss was calculated as follows: (volume follow-up – volume baseline)/(volume baseline × time in years).

### APOE Status

Whole blood samples were collected at baseline for all subjects for APOE genotyping. Standard DNA extraction was performed using either 9-ml EDTA tubes (Sarstedt, Germany) or Oragene Saliva DNA Kit (DNA Genotek, Inc., Ottawa, ON, Canada), which were stored at −20°C. APOE genotyping was done on the LightCycler (Roche Diagnostics, Basel, Switzerland) as described previously (35). Subjects were divided according to their APOE epsilon 4 allele status (4/3 vs. 3/3, 3/2 carriers).

### Statistics

Amyloid-positive and -negative cases were compared in respect to their clinical data with Fisher exact test, unpaired *t*-test, and Mann–Whitney *U*-test. Mixed effects linear regression models were used to identify predictors of the brain volume (dependent variable) including time, sex, age, personality factors (and facets), mean SUVR, APOE genotyping, and continuous cognitive score. The significance level was set at *p* < 0.05 but was corrected for multivariable testing by using the Benjamini–Hochberg method (36). All statistics were performed with the STATA statistical software, Version 16.1 (StataCorp, College Station, Texas, 2019).

## Results

### Descriptive Data

Men were overrepresented among amyloid-positive cases. Amyloid positivity was associated with significantly higher frequency of APOE epsilon 4 genotype, lower scores of Agreeableness, and increased mean SUVR (Table 1). The association between amyloid positivity and gender or lower Agreeableness did not survive after correction for multiple comparisons. Importantly, no case evolved to MCI during the follow-up period. To decrease the level of inter-individual variability, the mean SUVR (instead of binary amyloid classification) was used in further statistical analyses (37, 38).

### NEO-PI Factors

In univariate models, cases with lower Agreeableness scores displayed higher volumes at follow-up in PCC, PC, and amygdala bilaterally. This was also the case for right mPFC in elders displaying lower Agreeableness and Conscientiousness (Table 2). The percentage of variability in volume loss explained by agreeableness scores was of 26.3% (left and right amygdala), 24.4% (left PC), 16.7% (left PCC), 27.4% (right mPFC), 24.4% (right PC), and 13.8% (right PCC). This percentage was of 10.1% for the association between lower Conscientiousness and right mPFC volume loss. When correction for multiple comparisons was applied, the associations persisted in all of the abovementioned areas, except the right mPFC for Agreeableness.

**Table 2:** Association between mentalizing network component volume by side and personality dimensions assessed with univariate and multiple mixed linear regression, adjusted for time, sex, APOE4, amyloid1 load, and change in cognition.

		**Left side**						**Right side**					
		**Univariate**			**Multiple**			**Univariate**			**Multiple**		
**Brain region**	**Personality dimensions**	**Coeff (95% CI)**	***p***	**BH**	**Coeff (95% CI)**	***p***	**BH**	**Coeff (95% CI)**	***p***	**BH**	**Coeff (95% CI)**	***p***	**BH**
Medial prefrontal cortex (mPFC)	Neuroticism	−0.002 (−0.006, 0.001)	0.246		−0.002 (−0.006, 0.001)	0.139		−0.001 (−0.005, 0.002)	0.469		−0.002 (−0.005, 0.002)	0.380	
	Extraversion	0.002 (−0.003, 0.006)	0.433		0.002 (−0.003, 0.006)	0.435		0.001 (−0.004, 0.005)	0.757		−0.000 (−0.005, 0.004)	0.834	
	Openness	0.004 (0.000, 0.008)	0.039		0.004 (0.000, 0.008)	0.032		0.001 (−0.003, 0.005)	0.733		−0.001 (−0.004, 0.003)	0.800	
	Agreeableness	−0.000 (−0.004, 0.003)	0.871		0.002 (−0.001, 0.006)	0.200		−0.005 (−0.008, −0.001)	0.012		−0.004 (−0.007, 0.000)	0.068	
	Conscientiousness	0.001 (−0.003, 0.005)	0.647		−0.000 (−0.004, 0.004)	0.955		−0.005 (−0.009, −0.002)	0.005	^*^	−0.006 (−0.010, −0.003)	0.001	^*^
Posterior cingulate cortex (PCC)	Neuroticism	−0.001 (−0.009, 0.008)	0.835		−0.001 (−0.009, 0.006)	0.740		0.002 (−0.006, 0.010)	0.677		0.001 (−0.006, 0.008)	0.764	
	Extraversion	0.008 (−0.002, 0.018)	0.119		0.006 (−0.003, 0.016)	0.171		0.004 (−0.006, 0.014)	0.430		0.002 (−0.008, 0.011)	0.750	
	Openness	0.009 (−0.000, 0.018)	0.054		0.007 (−0.001, 0.015)	0.087		0.006 (−0.003, 0.015)	0.163		0.006 (−0.003, 0.014)	0.187	
	Agreeableness	−0.014 (−0.023, −0.006)	0.000	^*^	−0.008 (−0.016, 0.000)	0.061		−0.012 (−0.020, −0.004)	0.004	^*^	−0.005 (−0.014, 0.003)	0.195	
	Conscientiousness	−0.007 (−0.017, 0.002)	0.132		−0.011 (−0.018, −0.003)	0.009		−0.006 (−0.015, 0.003)	0.213		−0.009 (−0.017, −0.001)	0.022	
Precuneus	Neuroticism	−0.011 (−0.027, 0.006)	0.200		−0.010 (−0.024, 0.004)	0.154		−0.008 (−0.025, 0.009)	0.364		−0.008 (−0.022, 0.006)	0.253	
	Extraversion	0.007 (−0.013, 0.028)	0.474		0.003 (−0.015, 0.021)	0.740		0.012 (−0.009, 0.033)	0.253		0.011 (−0.007, 0.029)	0.236	
	Openness	−0.005 (−0.024, 0.013)	0.561		−0.005 (−0.021, 0.010)	0.502		−0.001 (−0.020, 0.018)	0.894		−0.001 (−0.018, 0.015)	0.880	
	Agreeableness	−0.035 (−0.050, −0.020)	0.000	^*^	−0.022 (−0.037, −0.007)	0.005		−0.030 (−0.047, −0.013)	0.000	^*^	−0.014 (−0.030, 0.002)	0.095	
	Conscientiousness	−0.008 (−0.026, 0.011)	0.411		−0.015 (−0.030, −0.000)	0.050		−0.006 (−0.025, 0.014)	0.571		−0.014 (−0.030, 0.002)	0.089	
Temporoparietal junction (TPJ)	Neuroticism	−0.012 (−0.027, 0.003)	0.106		−0.013 (−0.026, 0.000)	0.057		−0.006 (−0.023, 0.012)	0.527		−0.006 (−0.023, 0.010)	0.458	
	Extraversion	0.017 (−0.001, 0.035)	0.069		0.012 (−0.005, 0.030)	0.161		0.019 (−0.002, 0.040)	0.075		0.017 (−0.004, 0.039)	0.110	
	Openness	0.006 (−0.011, 0.022)	0.496		0.004 (−0.011, 0.020)	0.582		0.006 (−0.013, 0.025)	0.542		0.003 (−0.016, 0.023)	0.747	
	Agreeableness	−0.012 (−0.027, 0.004)	0.140		0.001 (−0.014, 0.017)	0.859		−0.009 (−0.028, 0.009)	0.319		0.002 (−0.017, 0.021)	0.844	
	Conscientiousness	−0.000 (−0.017, 0.017)	0.963		−0.005 (−0.020, 0.011)	0.556		0.001 (−0.019, 0.021)	0.947		−0.003 (−0.023, 0.016)	0.730	
Amygdala	Neuroticism	−0.001 (−0.002, 0.001)	0.300		−0.001 (−0.002, 0.000)	0.169		0.000 (−0.001, 0.002)	0.765		0.000 (−0.001, 0.001)	0.847	
	Extraversion	0.001 (−0.001, 0.003)	0.185		0.000 (−0.001, 0.002)	0.665		0.001 (−0.000, 0.003)	0.137		0.001 (−0.001, 0.002)	0.467	
	Openness	0.002 (0.000, 0.003)	0.046		0.001 (−0.000, 0.002)	0.143		0.001 (−0.001, 0.003)	0.199		0.000 (−0.001, 0.002)	0.916	
	Agreeableness	−0.003 (−0.004, −0.002)	0.000	^*^	−0.002 (−0.003, −0.000)	0.014		−0.003 (−0.004, −0.001)	0.000	^*^	−0.002 (−0.003, −0.000)	0.011	
	Conscientiousness	0.000 (−0.002, 0.002)	0.779		−0.000 (−0.002, 0.001)	0.755		0.000 (−0.001, 0.002)	0.597		0.000 (−0.001, 0.002)	0.858	
Caudate	Neuroticism	−0.000 (−0.005, 0.005)	0.916		−0.000 (−0.005, 0.005)	0.979		−0.000 (−0.005, 0.005)	0.948		0.000 (−0.005, 0.005)	0.942	
	Extraversion	0.004 (−0.002, 0.010)	0.168		0.002 (−0.004, 0.008)	0.471		0.005 (−0.001, 0.011)	0.119		0.003 (−0.003, 0.009)	0.404	
	Openness	0.003 (−0.002, 0.008)	0.258		0.002 (−0.003, 0.007)	0.412		0.005 (−0.000, 0.010)	0.074		0.003 (−0.002, 0.009)	0.208	
	Agreeableness	−0.004 (−0.009, 0.001)	0.140		−0.001 (−0.006, 0.004)	0.676		−0.002 (−0.007, 0.003)	0.519		0.001 (−0.005, 0.006)	0.773	
	Conscientiousness	−0.002 (−0.008, 0.003)	0.392		−0.003 (−0.008, 0.002)	0.210		−0.003 (−0.009, 0.002)	0.222		−0.004 (−0.009, 0.001)	0.113	
Fusiform gyrus	Neuroticism	−0.010 (−0.021, 0.002)	0.111		−0.009 (−0.019, 0.001)	0.080		−0.007 (−0.020, 0.006)	0.287		−0.006 (−0.017, 0.006)	0.330	
	Extraversion	0.004 (−0.011, 0.018)	0.616		0.001 (−0.012, 0.015)	0.863		0.001 (−0.015, 0.017)	0.892		−0.002 (−0.017, 0.013)	0.799	
	Openness	0.009 (−0.004, 0.022)	0.184		0.008 (−0.004, 0.020)	0.175		0.007 (−0.007, 0.021)	0.344		0.006 (−0.008, 0.019)	0.405	
	Agreeableness	−0.007 (−0.020, 0.005)	0.239		0.005 (−0.006, 0.017)	0.370		−0.015 (−0.029, −0.002)	0.026		−0.004 (−0.017, 0.010)	0.585	
	Conscientiousness	−0.003 (−0.016, 0.011)	0.709		−0.008 (−0.020, 0.003)	0.171		−0.003 (−0.018, 0.011)	0.648		−0.009 (−0.022, 0.004)	0.182	
Cerebellum	Neuroticism	−0.036 (−0.107, 0.034)	0.314		−0.033 (−0.097, 0.030)	0.303		−0.031 (−0.108, 0.045)	0.424		−0.027 (−0.095, 0.042)	0.447	
	Extraversion	0.021 (−0.067, 0.109)	0.643		−0.004 (−0.086, 0.079)	0.933		0.018 (−0.077, 0.113)	0.710		−0.013 (−0.102, 0.076)	0.779	
	Openness	−0.016 (−0.095, 0.064)	0.697		−0.035 (−0.108, 0.038)	0.350		−0.021 (−0.107, 0.064)	0.625		−0.044 (−0.123, 0.034)	0.270	
	Agreeableness	−0.054 (−0.129, 0.020)	0.154		0.015 (−0.059, 0.088)	0.699		−0.065 (−0.145, 0.015)	0.112		0.008 (−0.072, 0.087)	0.852	
	Conscientiousness	−0.001 (−0.084, 0.081)	0.972		−0.024 (−0.097, 0.049)	0.523		−0.007 (−0.096, 0.082)	0.874		−0.030 (−0.109, 0.048)	0.448	

*p values are uncorrected. The Benjamini–Hochberg threshold is p = 0.005 for the univariate and p = 0.001 for the multivariable analysis*.

* *Indicates significant values according to Benjamini-Hochberg correction*.

In multiple regression models, the negative association between Agreeableness and brain volume was still observed in left PC (38.4% of variability, significant sex variable) and left (35.6% of variability, significant sex and APOE 4 genotype variables) and right amygdala (30.4% of variability, significant sex, APOE4, mean SUVr, and change of continuous cognitive score). This was also the case for the negative association between Conscientiousness and right mPFC volume (26.3% of variability, significant age and sex). Interestingly, a significant association emerged in multivariate models between higher Conscientiousness and increased brain volume loss in bilateral PCC and left PC (Table 2). After correction for multiple comparisons, the significance was preserved for the association between higher Agreeableness scores and increased volume loss in left PC. This was also the case for the association between higher Conscientiousness scores and increased volume loss in right mPFC. There were no associations between NEO-PI factors and brain volume changes in all of the control areas.

### NEO-PI Facets

The NEO-PI facets of Agreeableness and Conscientiousness have also been considered in regression models. We retained only the associations that survived in multiple regression models and after correction for multiple comparisons (Tables 3, 4). Among agreeableness facets, higher modesty scores were associated with increased volume loss in left PC and right mPFC. Conscientiousness facets had also a negative association with brain volumes within the mentalizing network. In the left hemisphere, higher-order scores (C2) were related to decreased PCC volume at follow-up. In the right hemisphere, the same facet scores were negatively related to PC and PCC volumes. Self-discipline scores were negatively related to PCC volumes. Most importantly, order, dutifulness, achievement striving, and self-discipline scores were all negatively related to mPFC volume at follow-up. In all of the control areas, no association was found between NEO-PI personality facets and volume loss.

**Table 3:** Association between mentalizing network component volume by side and facets of Agreeableness assessed with multiple mixed linear regression, adjusted for time, sex, APOE4, amyloid load, and change in cognition.

			**Left side**			**Right side**	
**Brain region**	**Facets**	**Coeff (95% CI)**	***p***	**BH**	**Coeff (95% CI)**	***p***	**BH**
Medial prefrontal cortex (mPFC)	Trust	0.014 (−0.002, 0.030)	0.096		−0.001 (−0.018, 0.016)	0.906	
	Straightforwardness/morality	0.006 (−0.007, 0.020)	0.363		−0.011 (−0.025, 0.003)	0.134	
	Altruism	0.004 (−0.016, 0.024)	0.709		−0.014 (−0.034, 0.006)	0.168	
	Compliance/cooperation	0.005 (−0.012, 0.022)	0.572		−0.005 (−0.023, 0.012)	0.547	
	Modesty	−0.001 (−0.015, 0.013)	0.871		−0.020 (−0.034, −0.007)	0.003	^*^
	Tendermindedness/sympathy	0.024 (0.006, 0.043)	0.011		−0.009 (−0.029, 0.011)	0.379	
Posterior cingulate cortex (PCC)	Trust	−0.006 (−0.043, 0.030)	0.742		−0.011 (−0.048, 0.025)	0.547	
	Straightforwardness/morality	−0.017 (−0.047, 0.012)	0.250		−0.020 (−0.050, 0.010)	0.191	
	Altruism	−0.055 (−0.096, −0.013)	0.010		−0.035 (−0.078, 0.007)	0.101	
	Compliance/cooperation	−0.024 (−0.060, 0.012)	0.199		−0.004 (−0.041, 0.033)	0.838	
	Modesty	−0.023 (−0.053, 0.006)	0.120		−0.010 (−0.040, 0.019)	0.496	
	Tendermindedness/sympathy	−0.028 (−0.071, 0.014)	0.190		−0.025 (−0.068, 0.017)	0.244	
Precuneus	Trust	−0.047 (−0.115, 0.022)	0.184		−0.014 (−0.085, 0.057)	0.703	
	Straightforwardness/morality	−0.044 (−0.100, 0.012)	0.124		−0.038 (−0.096, 0.020)	0.202	
	Altruism	−0.089 (−0.168, −0.010)	0.026		−0.062 (−0.145, 0.022)	0.151	
	Compliance/cooperation	−0.025 (−0.094, 0.044)	0.476		−0.019 (−0.090, 0.053)	0.606	
	Modesty	−0.100 (−0.151, −0.049)	<0.001	^*^	−0.062 (−0.119, −0.006)	0.030	
	Tendermindedness/sympathy	−0.078 (−0.158, 0.002)	0.055		−0.047 (−0.130, 0.036)	0.268	
Amygdala	Trust	−0.005 (−0.011, 0.002)	0.149		−0.006 (−0.012, 0.000)	0.056	
	Straightforwardness/morality	−0.004 (−0.009, 0.001)	0.102		−0.007 (−0.012, −0.002)	0.011	
	Altruism	−0.005 (−0.013, 0.002)	0.166		−0.004 (−0.011, 0.004)	0.345	
	Compliance/cooperation	−0.008 (−0.014, −0.002)	0.007		−0.006 (−0.012, 0.000)	0.058	
	Modesty	−0.004 (−0.009, 0.001)	0.146		−0.004 (−0.009, 0.001)	0.145	
	Tendermindedness/sympathy	−0.006 (−0.013, 0.001)	0.096		−0.006 (−0.013, 0.001)	0.110	

*p values are uncorrected. The Benjamini–Hochberg threshold is p = 0.005*.

* *Indicates significant values according to Benjamini-Hochberg correction*.

**Table 4:** Association between mentalizing network component volume by side and facets of Conscientiousness assessed with multiple mixed linear regression, adjusted for time, sex, APOE4, amyloid load, and change in cognition.

			**Left side**			**Right side**	
**Brain region**	**Facets**	**Coeff (95% CI)**	***p***	**BH**	**Coeff (95% CI)**	***p***	**BH**
Medial prefrontal cortex (mPFC)	Competence	0.006 (−0.012, 0.024)	0.506		−0.007 (−0.026, 0.012)	0.472	
	Order	−0.005 (−0.019, 0.010)	0.553		−0.020 (−0.034, −0.005)	0.007	^*^
	Dutifulness	−0.002 (−0.019, 0.016)	0.868		−0.025 (−0.042, −0.008)	0.003	^*^
	Achievement striving	0.004 (−0.017, 0.025)	0.728		−0.038 (−0.058, −0.019)	0.000	^*^
	Self–discipline	−0.004 (−0.018, 0.010)	0.554		−0.023 (−0.036, −0.010)	0.001	^*^
	Deliberation	0.005 (−0.015, 0.025)	0.623		−0.017 (−0.037, 0.003)	0.093	
Posterior cingulate cortex (PCC)	Competence	−0.031 (−0.070, 0.008)	0.123		−0.035 (−0.074, 0.004)	0.075	
	Order	−0.048 (−0.078, −0.017)	0.002		−0.043 (−0.074, −0.012)	0.007	^*^
	Dutifulness	−0.026 (−0.064, 0.012)	0.185		−0.012 (−0.050, 0.026)	0.538	
	Achievement striving	−0.027 (−0.073, 0.019)	0.256		−0.011 (−0.058, 0.035)	0.635	
	Self–discipline	−0.041 (−0.069, −0.013)	0.004		−0.043 (−0.071, −0.015)	0.003	^*^
	Deliberation	−0.033 (−0.075, 0.010)	0.136		−0.023 (−0.066, 0.020)	0.301	
Precuneus	Competence	0.007 (−0.068, 0.083)	0.855		−0.037 (−0.115, 0.040)	0.342	
	Order	−0.078 (−0.136, −0.020)	0.009		−0.083 (−0.143, −0.023)	0.007	^*^
	Dutifulness	−0.013 (−0.085, 0.059)	0.721		−0.000 (−0.076, 0.075)	0.990	
	Achievement striving	−0.071 (−0.157, 0.015)	0.107		−0.044 (−0.134, 0.046)	0.334	
	Self–discipline	−0.068 (−0.121, −0.014)	0.014		−0.056 (−0.113, 0.001)	0.054	
	Deliberation	−0.066 (−0.146, 0.014)	0.108		−0.027 (−0.112, 0.057)	0.528	
Amygdala	Competence	0.004 (−0.003, 0.011)	0.282		0.002 (−0.005, 0.009)	0.516	
	Order	−0.002 (−0.008, 0.003)	0.430		−0.000 (−0.006, 0.006)	0.974	
	Dutifulness	−0.002 (−0.008, 0.005)	0.626		0.001 (−0.006, 0.008)	0.742	
	Achievement striving	0.001 (−0.007, 0.009)	0.898		0.004 (−0.004, 0.012)	0.275	
	Self-discipline	−0.002 (−0.007, 0.003)	0.475		−0.002 (−0.007, 0.003)	0.444	
	Deliberation	−0.001 (−0.009, 0.006)	0.774		−0.000 (−0.008, 0.007)	0.912	

*p values are uncorrected. The Benjamini–Hochberg threshold is p = 0.005 (left side) and p = 0.008 (right side)*.

* *Indicates significant values according to Benjamini-Hochberg correction*.

## Discussion

The present findings reveal that higher levels of Agreeableness and Conscientiousness have a negative impact on the structural integrity of the mentalizing network. This observation concerned not only the factors but also the corresponding facets (modesty for Agreeableness and order, dutifulness, achievement striving, and self-discipline for Conscientiousness). The impact of these personality factors was mainly present in mPFC, PC, and PCC, the three main cortical components of the mentalizing network as well as in amygdala but not in TPJ. Pointing to the specificity of our findings, such associations were not found in control areas (caudate, fusiform gyrus, and cerebellum) and did not concern the other personality factors.

Early cross-sectional data on the association between NEO-PI factors (and facets) and MRI volumes in brain aging revealed discrepant findings. Lower Openness scores were related to a widespread decrease of gray matter volumes whereas higher Neuroticism and scores were associated with decreased volumes in frontal and temporal cortices. Extraversion and Agreeableness scores display positive associations with superior, medial, and orbitofrontal cortex volumes, whereas the effect of Conscientiousness is more ambiguous (36–38). In a recent longitudinal study, we reported that lower Agreeableness and higher Openness are associated with better preservation of the areas early affected by Alzheimer disease pathology such as mesial temporal lobe and hippocampus (25). In particular and unlike functional imaging data on DMN and our own observations in AD-related areas (11–14, 25), Openness to experience scores were unrelated to the rate of volume loss in mentalizing network. Taken together, these observations did not support a global effect of personality factors (and facets) on brain aging processes but rather suggests that they have differential impact on brain integrity depending on the circuits studied.

The clinical significance of the present findings merits further development. Traditionally, high Agreeableness in adult lifespan is thought to be a positive trait of personality being associated with increased subjective well-being (39), better outcome in mental health treatments (40), less disengagement coping (41), and less sexual aggressive behavior (42). In old age, higher Agreeableness levels have been instead associated with poorer executive performance and neurocognitive functions (43–45) and medically unexplained symptoms (46). The role of higher levels of Conscientiousness in old age is equally ambiguous. They were associated with more positive attitudes toward own aging (47), increased well-being (48), and more favorable biomedical markers of health status (49) but also increased late-onset suicide attempts (50), decreased benefit of mental demands at workplace (51), and increased exposure to mental health problems (52). Unlike Neuroticism, high levels of Agreeableness and Conscientiousness were frequently considered as positive characteristics in the course of adult life. Agreeable persons are more prone to establish interpersonal relationships without aggressiveness searching for social approval adopting a majoritarian viewpoint. In old age, this kind of social adaption to other's willingness may be much less imperative. Conscientiousness corresponds to the individual ability to regulate impulsiveness and adopt a stable and rational communication style. Individuals with a high level of conscientiousness may formulate long-range goals, being able to work consistently to achieve them. In adult life, they may be seen as responsible and reliable persons. However, when work is less present in daily life, they may be seen as compulsive perfectionists, boring, or with rigid defense mechanisms. The present findings indicate that higher levels of Agreeableness and Conscientiousness may be detrimental for the structural integrity of the mentalizing network since they are associated with increasing rate of atrophy of some of its main components. In our series, higher agreeableness was also related to amyloid positivity, further supporting the idea that, unlike young age, it may represent a factor associated with brain vulnerability in old age (25).

Some strengths of the present work should be discussed. Volume loss in old age is a multifactorial phenomenon that is determined by demographic parameters (age, gender), genetic predisposal (in particular APOE epsilon 4 genotype), and progressive formation of aging-related pathologies such as vascular lesions and amyloid accumulation. Moreover, the variability of cognitive trajectories in elderly persons is an additional confounder that correlates with brain volume changes over time in elderly individuals. In a community-based cohort with careful exclusion of significant vascular burden, psychiatric and neurological conditions, and drug abuse, we had the opportunity to control for the relative contribution of all of the previously mentioned factors. The second issue concerns the obvious risk of multiple comparison biases when assessing the relationship between NEO-PI personality factors (and facets) and volumes of various brain areas. To limit this risk, we first formulated a priori hypotheses focusing on the mentalizing network. In addition, the association between personality and MRI measures was studied using a stringent criterion for multiple comparisons to exclude false-positive results. This is particularly important in respect to the numerous personality facets that have been taken into account. Four main limitations should be considered when interpreting these observations. First, baseline and follow-up MRI were acquired on two different scanners, due to the longitudinal study design. One could speculate that this change could confound the estimated volumes. The change of MR scanners is a known problem in a clinical setting. We carefully matched the MR sequences between both scanners and used software with compensation algorithms. Most importantly, our regression models aim to explore the association between personality factors (and facets) and brain volume changes. They are thus not affected by MRI scan changes as could be the cases when assessing group differences. Second, our cases show no or very mild vascular pathology and relatively high level of education. Although necessary for controlling the confounding effect of this variable, this way to proceed decreases the representativeness of our sample. Third, the combination of all significant predictors allows for explaining <40% of the volume loss variability in the areas studied. Although substantial in the light of the marked heterogeneity of normal aging and relatively small sample size, this percentage indicates the presence of additional predictors that have been not taken into account in our analysis. Finally, this study focuses on volumetric changes and did not include a functional MRI component. We cannot thus comment on the association between personality and patterns of functional activation within the mentalizing network in old age.

In conclusion, we report here a specific association between lower levels of Agreeableness and Conscientiousness and better preservation of the volume of mentalizing network components in old age. In the light of these findings, one could speculate that ToM performances may be more resistant in the subsample of cognitively preserved elders with such NEO-PI profile. Although research on the association between personality factors and mentalizing is still in its infancy, some first data point to the idea that at least some components of Agreeableness may be negatively associated with this main human ability (15). Future studies in larger community-based cohorts including *ad hoc* theory of mind activation paradigms tasks, as well as *in vivo* assessment of tau pathology and brain metabolism, are warranted to further explore the role of personality in age-related changes of the mentalizing network.

## Data Availability Statement

The original contributions presented in the study are publicly available. This data can be found at: https://doi.org/10.5061/dryad.3tx95x6dp.

## Ethics Statement

The studies involving human participants were reviewed and approved by Commission cantonale d'éthique de la recherche CCER. The patients/participants provided their written informed consent to participate in this study.

## Author Contributions

PG and FH contributed to the study design and wrote the paper. CR and VG were involved in participant recruitment, follow-up, and data acquisition. M-LM, SH, FH, and PG performed and analyzed the data. All authors read and approved the final manuscript.

## Conflict of Interest

The authors declare that the research was conducted in the absence of any commercial or financial relationships that could be construed as a potential conflict of interest.
